# Comparison of the Transformation Ability of the Major Saponins in *Panax notoginseng* by *Penicillum fimorum* Enzyme and Commercial *β*-glucosidase

**DOI:** 10.3390/microorganisms13030495

**Published:** 2025-02-23

**Authors:** Feixing Li, Ruixue Zhang, Dongmei Lin, Jin Yang, Ye Yang, Xiuming Cui, Xiaoyan Yang

**Affiliations:** 1 Faculty of Life Science and Technology, Kunming University of Science and Technology, Kunming 650500, China; 20222218039@stu.kust.edu.cn (F.L.); 20232118004@stu.kust.edu.cn (R.Z.); 20222118071@stu.kust.edu.cn (D.L.); 20232118083@stu.kust.edu.cn (J.Y.); yangye@kust.edu.cn (Y.Y.); 20120094@kust.edu.cn (X.C.); 2 Yunnan Key Laboratory of Sustainable Utilization of Panax Notoginseng, Kunming 650500, China

**Keywords:** *Panax notoginseng* root, biotransformation, *Penicillum fimorum*, minor ginsenosides

## Abstract

Ginsenosides with less sugar groups, which are called minor ginsenosides, might have a greater pharmacological activity and better adsorptive ability, but their content in nature is extremely low. In this study, a strain of *Penicillium fimorum* with a strong saponin transformation ability was isolated from fresh *Gastrodia elata*. A comparative biotransformation experiment of the major saponins from *Panax notoginseng* root were conducted using crude enzymes from *P. fimorum* and commercial *β*-glucosidase to produce minor ginsenosides. Specifically, the crude enzyme from *P. fimorum* was able to transform the major saponins from *P. notoginseng* root into 13 minor saponins in 72 h, while commercial *β*-glucosidase was able to transform the same major saponins into 15 minor saponins in 72 h. The most significant difference between these two enzymes is their ability to transform Rb_1_. To the best of our knowledge, the biotransformation ability of crude enzymes from *P. fimorum* is reported here for the first time. These two enzymes have the potential to improve the economic value of *P. notoginseng* root and expand the methods for preparing minor saponins by transforming major saponins in the total saponins of *P. notoginseng* root.

## 1. Introduction

The genus of *Panax* belongs to the Araliaceae family. There are 17 species of the *Panax* genus, including *P. ginseng*, *P. quinquefolius*, and *P. notoginseng* [1]. Most members of this genus have medicinal properties and more than 150 ginsenosides have been identified and classified according to their structures, which can be divided into damarane, oktylon, and oleanolic acid types [2,3]. *P. notoginseng* (Burk.) F. H. Chen is a traditional and valuable Chinese herbal medicine, mainly grown in Guangxi and Yunnan Provinces in Southwest China. In *P. notoginseng*, dalmarane-type tetracyclic triterpene saponins can be further divided into propanaxadiol-type saponins (PPD, such as Rb_1_, Rb_2_, Rb_3_, Rd, and Rc) and propanaxtriol-type saponins (PPT, such as Re and Rg_1_) according to the presence of a hydroxyl group attached to C-6. The types and contents of saponins in different parts of *P. notoginseng* are also different, and *P. notoginseng* root (PNR) mainly contains PPD-type ginsenosides (Rb_1_ and Rd) and PPT-type ginsenosides (Re, Rg_1_, and R_1_) [4]. The major saponins in PNR mainly show great polarity because of the different types and amounts of sugar moieties linked to the C-3, C-6, and C-20 positions of the dammarane triterpenoid backbone [5]. After oral administration, the major saponins need to be hydrolyzed by digestive enzymes and intestinal microorganisms before they can become more active and easily absorbed minor ginsenosides, but the efficiency of these conversions is very low [6].

Most studies have shown that the function of ginsenosides is closely related to the position, type, and quantity of glycosidic bonds [7]. The main ginsenosides contain more sugar groups, which leads to their low pharmacological activity, and they are not easily absorbed by the human body. Compared with the main ginsenosides, minor ginsenosides have better pharmacological activities, such as anti-hypertension and anti-aging [8]. These minor saponins are produced mainly by deglycosylation, isomerization, and dehydration [9,10]. The main ginsenosides can be hydrolyzed into minor ginsenosides through physical, chemical, and biotransformation. Compared with other methods, biotransformation has the advantages of mild conversion conditions, product stability, and being pollution-free [11].

In order to prepare minor ginsenosides, many microorganisms capable of transforming ginsenosides have been discovered and applied. In particular, the relatively safe strain of *Aspergillus niger* is well known. *A. niger* JGL8 isolated from *Gynostemma pentaphyllum* can transform *Gynostemma pentaphyllum* saponins to ginsenoside F_2_ through GypV →Rd→F_2_ [12]. Ginsenoside Rb_1_ can be transformed by extracellular enzymes produced by *A. niger* WU-16. The transformation pathway of Rb_1_ is Rb_1_→F_2_, CK [13]. *A. Niger* XD101 and its crude enzymes screened from the planting soil of *P. notoginseng* also have the ability to hydrolyze ginsenoside Rb_1_, and its transformation pathway is as follows: Rb_1_→Rd→F_2_→CK [14]. The results of these studies indicate the feasibility of using microorganisms to carry out the transformation of major saponins for the preparation of minor saponins. Moreover, previous studies have shown there are four main enzymes used in the enzymatic production of minor ginsenosides. These four enzymes hydrolyze glycosidic bonds at different positions on major ginsenosides [15,16,17,18,19,20].

In this study, a strain of the plant endophytic fungus *P. fimorum* with an excellent saponin transformation capacity was isolated from fresh *Gastrodia elata*, and its extracellular crude enzyme was extracted to transform several major saponins in PNR for 72 h. The enzyme was found to be capable of transforming four major saponins (Rb_1_, Rg_1_, R_1_, and Re) in PNR into 13 minor saponins, of which ginsenoside CK had the highest yield at 11.88%, followed by ginsenoside Rd with a yield of 13.03%. To the best of our knowledge, this biotransformation characteristic of *P. fimorum* is reported here for the first time in fungi. In addition, commercial *β*-glucosidase was purchased for comparison, and it was found to transform the four major saponins in PNR into 15 minor saponins, of which ginsenoside Rd had the highest yield at 53.87%. The yields of the remaining products were similar to those obtained from transformation through the extracellular crude enzyme of *P. fimorum*.

By analyzing two comparative experiments, we found that the extracellular crude enzyme of *P. fimorum* and commercial *β*-glucosidase exhibited the following transformation capacities: deglycosylation, epimerization, and dehydration. The main difference is that the extracellular crude enzyme of *P. fimorum* specifically hydrolyzes glucose at the C-3 position of ginsenoside Rb_1_, efficiently producing ginsenoside CK, whereas *β*-glucosidase specifically hydrolyzes glucose at the C-20 position of ginsenoside Rb_1_, producing ginsenosides Rd, 20(*S*/*R*)-Rg_3_, Rk_1_, and Rg_5_. Through an analysis of the dynamic changes in the content of substrates and products during the transformation process, we deduced the transformation pathways of four major saponins mediated by the *P. fimorum* enzyme and commercial *β*-glucosidase, and calculated the substrate conversion rates and product yields. This study is expected to facilitate the conversion of total saponins from PNR into minor saponins, thereby greatly enhancing the economic and functional value of *P. notoginseng*.

## 2. Materials and Methods

### 2.1. Materials

Authentic standards of ginsenosides Rb_1_, Rd, F_2_, CK, 20(*S*/*R*)-Rg_3_, Rk_1_, Rg_5_, 20(*S*/*R*)-Rh_1_, Rh_4_, Rk_3_, Re, 20(*S*/*R*)-Rg_2_, F_4_, Rg_6_, and notoginsenosides R_1_, 20(*S*/*R*)-R_2_ were purchased from the Sichuan Victory, with purities of HPLC ≥ 98%. The commercial *β*-glucosidase (CAS: 90001-22-3) was purchased from Shanghai Yuanye Bio-Technology Co., Ltd. (Shanghai, China). The solvents methanol, acetic acid, and trichloromethane of analytically pure grade (Nuoershi brand) for the TLC analysis were purchased from Chengdu Cologne Chemical Co. (Chengdu, China). The solvents methanol and acetonitrile for HPLC, which are of chromatographic pure grade, were purchased from Sigma-Aldrich Co. (St. Louis, MO, USA). The HSGF_254_ silica gel TLC plate was purchased from Yantai Jiangyou Silicone Development Co., Ltd. (Yantai, China). A Welchrom C_18_ column (4.6 × 250 mm, 5 µm) was sourced from Yuexu Technology Co., Ltd. (Mianyang, China). An Agilent 1260 high-performance liquid chromatography instrument was purchased from Agilent (Grand Island, NY, USA).

### 2.2. Medium

PDA medium: potato extract powder 6 g/L, glucose 25 g/L, and agar 25 g/L. PDB medium: potato extract powder 6 g/L and glucose 25 g/L.

### 2.3. Isolation and Identification of Plant Endophytic Fungi

The plant tissue isolation method was used for the isolation and purification of endophytes [21,22]. First, 500 g of fresh *Gastrodia elata* were collected, cleaned, and surface-sterilized. Then, multiple tissue slices (0.5 cm × 0.5 cm) were prepared and subsequently transferred onto PDA medium for constant temperature cultivation, from which the colonies were isolated, purified, and screened to obtain a single fungal colony, designated as S62. The amplification and sequencing of the ITS rDNA gene was performed by the Kunming Branch of Tsingke Biotechnology Co., Ltd. (Kunming, China). The ITS rDNA gene was submitted to the National Centre for Biotechnology Information (NCBI) to obtain the NCBI-recorded RSA registry number OR958833. The phylogenetic tree was constructed using the neighbor-joining (NJ) algorithm implemented in MEGA software (version 11.0). The morphologies of conidiophores and ascospores were observed through optical microscopy. A voucher specimen (No. Li20221001) was deposited at the Faculty of Life Science and Technology, Kunming University of Science and Technology.

### 2.4. Screening of Fungi for Its Ability to Biotransform Saponins

Microbial fermentation of the fungus was carried out using PDB medium. After 5 days of fermentation, a solution of total saponins from PNR was added to the fermentation broth to continue microbial transformation for an additional 18 days. The total saponins solution of PNR was dissolved in 75% ethanol and filtered through a 0.22 μm filter before being added to the fermentation broth, achieving a final concentration of 4 mg/mL in the broth. After 18 days of fermentation, the fermentation broth was extracted with an equal volume (1:1) of aqueous-saturated n-BuOH. The developing solvent for the TLC analysis was CHCl_3_−CH_3_COOH−CH_3_OH−H_2_O (6.75:0.25:2.5:0.5, *v*/*v*/*v*/*v*). TLC was performed on silica gel plates. The spots on TLC were visualized by UV light (254/365 nm) and sprayed with 10% H_2_SO_4_ in ethanol, followed by heating. We assessed the saponin conversion capacity using the developed points between the conversion products and the substrate and minor saponin standards [23,24].

### 2.5. Preparation of Crude Enzyme from Microorganism

The extracellular crude enzymes of *P. fimorum* were extracted using supersaturated ammonium sulfate [4,25]. The fermentation broth from the 18-day fermentation was extracted with aqueous-saturated n-BuOH. Ammonium sulfate was then added to the lower phase of the fermentation broth to form a saturated solution, followed by the extraction of the crude enzyme.

### 2.6. Determination of Optimal pH and Optimal Temperature

Optimization of the optimal conditions for the enzyme reaction was carried out using ginsenoside Rb_1_. A 0.5 mL aliquot of 1 mg/mL ginsenoside Rb_1_ and 1 mL of 4 mg/mL enzyme solution were mixed well, and the reaction was then carried out. The buffer solution was prepared using phosphate and citrate with a pH range of 3–8. The reaction temperature was varied between 30–70 °C.

### 2.7. Biotransformation of Monomer Saponins by the P. fimorum Enzyme and Commercial β-glucosidase

Enzymatic biotransformation of four monomeric saponins (Rb_1_, Rg_1_, Re, and R_1_) from PNR was carried out for 72 h under optimal reaction conditions. At the end of the reaction, the solution was extracted with twice the volume of aqueous-saturated n-BuOH. The upper solution was then evaporated to dryness and analyzed using TLC and HPLC.

### 2.8. Monitoring of Substrate Conversion and Product Yields

To monitor changes in the substrate and products during biotransformation by the enzyme, samples from different time points were collected. Enzyme–substrate conversion reactions were performed at 3, 6, 12, 24, 48, and 72 h, respectively. At the end of the reaction, the solution was extracted with twice the volume of aqueous-saturated n-BuOH. The upper solution was then evaporated to dryness and analyzed using TLC and HPLC.

(1) $$Substrate\;conversion\;(\%)=\frac{C_{s}\;\left(\mathrm{mg}\right)-C_{fs}\;(\mathrm{mg})}{C_{s}\;\left(\mathrm{mg}\right)}$$

(2) $$Yield\;of\;product\;(\%)=\frac{\;C_{p}\;\left(\mathrm{mg}\right)\;}{{\;C}_{s}\;\left(\mathrm{mg}\right)\;}$$

where *C_s_* is the initial concentration of the substrate, *C_fs_* is the final concentration of the substrate, and *C_p_* is the final concentration of the product ginsenoside.

### 2.9. Analytical Methods of TLC and HPLC

Experimental samples were extracted using water-saturated n-butanol, and the upper solution was dried and used for subsequent experiments.

For the TLC analysis, CHCl_3_−CH_3_COOH−CH_3_OH−H_2_O (6.75:0.25:2.5:0.5, *v*/*v*/*v*/*v*) was used as a developing solvent, and 10% (*v*/*v*) H_2_SO_4_−ethanol was used as a chromogenic solvent.

For the HPLC analysis, the samples were analyzed by HPLC, using an Agilent 1260 system (Grand Island, NY, USA) connected to a Welchrom C_18_ chromatography column (4.6 × 250 mm, 5 µm). The mobile phase entailed a mixture of water (A) and acetonitrile (B). The gradient elution program was set as follows: 0−15 min (20% B), 15−20 min (20−30% B), 20−45 min (30−36% B), 45−52 min (36−38% B), 52−55 min (38−40% B), 55−63 min (40−42% B), 63−69 min (42−44% B), 69−75 min (44−51% B), 75−85 min (51−53% B), 85−90 min (53−60% B), 90−91 min (60−63% B), 91−100 min (63−66% B), and 100−105 min (66−100% B). The system maintained a flow rate of 1.0 mL/min. Absorbance measurements were taken at 203 nm with an injection volume of 30 μL and the column temperature was set at 25 °C. We identified different compounds by comparing the peak retention times of the transformation products, substrates, and minor saponin standards in HPLC.

## 3. Results

### 3.1. Screening and Characterisation of Strain for the Transformation of Saponins

**Figure 1:**
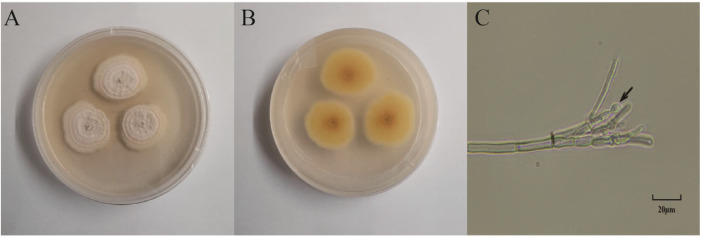
Morphology identification of strain S62. (**A**) Front view of the morphology of strain S62. (**B**) Back view of the morphology of strain S62. (**C**) Microscopic view of mycelium spore morphology of strain S62.

The plant endophytic fungus S62 was isolated from fresh *Gastrodia elata*. The morphology of the colonies after 5 days of culture on PDA medium is shown in Figure 1A,B. The surface appeared white, fluffy, and flocculent, while the back side was light yellow. Under optical microscopy, asymmetrical broom-like branches and subglobose conidia were observed.

**Figure 2:**
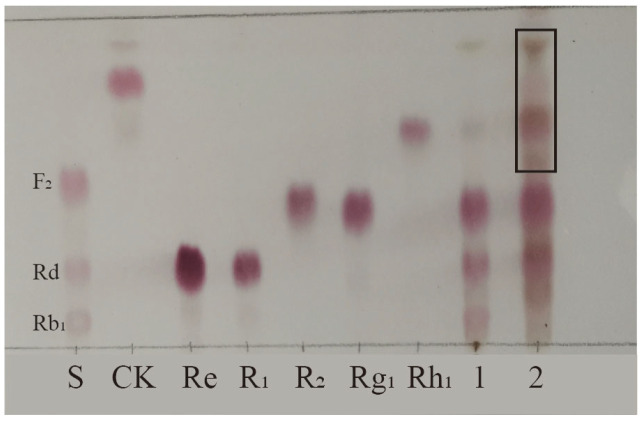
TLC analysis of the transformation products of total saponins from *P. notoginseng* root by *P. fimorum* for 18 days. S: Standards; 1: Substrate; 2: Transformation products after 18 days of biotransformation. Black square: Transformation products.

Biotransformation of the total saponins from PNR was carried out for 18 days using strain S62 in PDB medium. A TLC analysis of the results shown in Figure 2 reveals new spots with a larger Rf value above the original substrate, indicating that strain S62 has a better saponin conversion ability.

**Figure 3:**
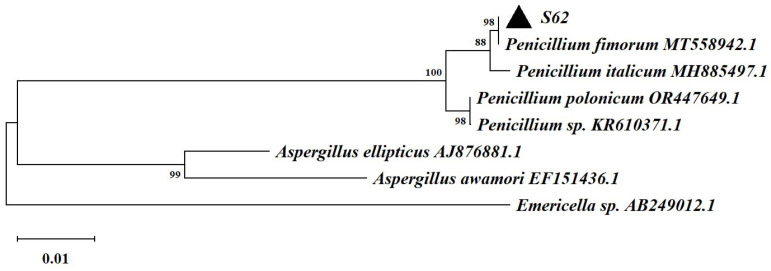
The neighbor-joining tree based on the ITS rDNA gene sequences of strain S62.

Based on the ITS rDNA gene sequencing and comparison with the GenBank database, strain S62 was found to belong to the genus *Penicillium* and exhibited remarkable similarity to *Penicillium fimorum*, as shown in Figure 3.

### 3.2. Results of Optimal pH and Temperature for Enzyme Transformation of Monomeric Saponin

**Figure 4:**
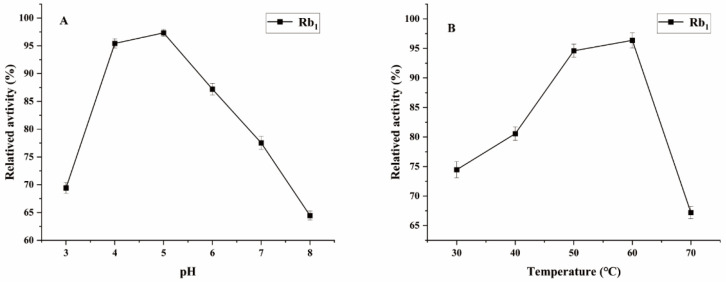
The optimum reaction temperature and pH for saponins transformed by the crude enzyme from *P. fimorum*. (**A**) pH effect on the transformation of Rb_1_. (**B**) Temperature effect on the transformation of Rb_1_.

**Figure 5:**
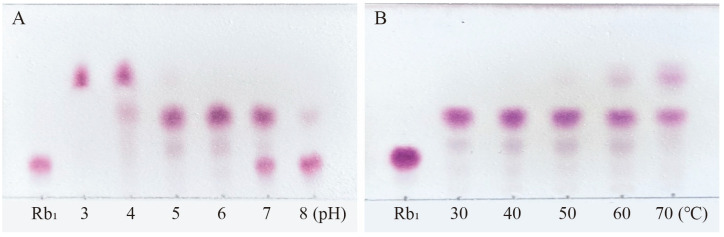
The optimum reaction temperature and pH for saponins transformed by *β*-glucosidase. (**A**) pH effect on the transformation of Rb_1_. (**B**) Temperature effect on the transformation of Rb_1_.

Ginsenoside Rb_1_, which represents the predominant saponin constituent in PNR with the highest concentration among common ginsenosides, was selected as the target compound for optimizing biotransformation parameters [13]. Figure 4 shows the optimal conditions for conversion by the *P. fimorum* crude enzyme, with an optimal pH of 5 and an optimal temperature of 60 °C. Similarly, the optimal reaction conditions for commercial *β*-glucosidase are also pH 5 and 60 °C, as shown in Figure 5. These results indicate that both enzymes exhibit optimal activity under weakly acidic conditions, which is similar to the enzyme reaction conditions reported for other strains with a saponin-conversion ability [26,27]. Both enzymes also exhibit good temperature tolerance, with activity remaining effective up to 60 °C. Additionally, a control experiment was conducted to examine the effects of pH and temperature on the reaction conditions. It was found that acidic hydrolysis of ginsenoside Rb_1_ occurred at both pH 3 and pH 4, while high-temperature pyrolysis was observed at 70 °C, as shown in Figure S1.

### 3.3. Transformation Ability of Enzymes on Monomer Ginsenosides Rb_1_, Rg_1_, Re, and Notoginsenoside R_1_

**Figure 6:**
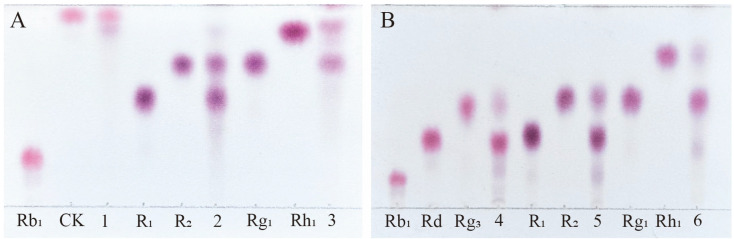
TLC analysis of the transformation products of different types of saponins (Rb_1_, R_1_, and Rg_1_) by crude enzymes from *P. fimorum* and *β*-glucosidase, respectively. (**A**) TLC analysis of the transformation products by the crude enzyme from *P. fimorum*. (**B**) TLC analysis of the transformation products by *β*-glucosidase. 1, 2, 3: The transformation products of Rb_1_, R_1_, and Rg_1_ by the crude enzyme from *P. fimorum*, respectively. 4, 5, 6: The transformation products of Rb_1_, R_1_, and Rg_1_ by *β*-glucosidase, respectively.

Among the total saponins of PNR, Rb_1_ is a PPD-type saponin, Rg_1_ is a PPT-type saponin, and R_1_ is also a PPT-type saponin, which is a type of saponin mainly contained in PNR [28]. Therefore, the enzyme’s ability to transform the total saponins of PNR can be indirectly inferred from its conversion of these three saponins. Figure 6A shows the TLC analysis of the crude enzyme transformation of these three types of saponins by *P. fimorum*, where ginsenoside Rb_1_ was almost completely transformed to ginsenoside CK, while ginsenoside Rg_1_ and notoginseng R_1_ were transformed to other minor saponins. Figure 6B presents the TLC results of the transformation of these three saponins by commercial *β*-glucosidase, where ginsenoside Rb_1_ was predominantly transformed into ginsenosides Rd and Rg_3_, while commercial *β*-glucosidase also transformed ginsenoside Rg_1_ and notoginseng R_1_, similar to the crude enzyme of *P. fimorum*.

**Figure 7:**
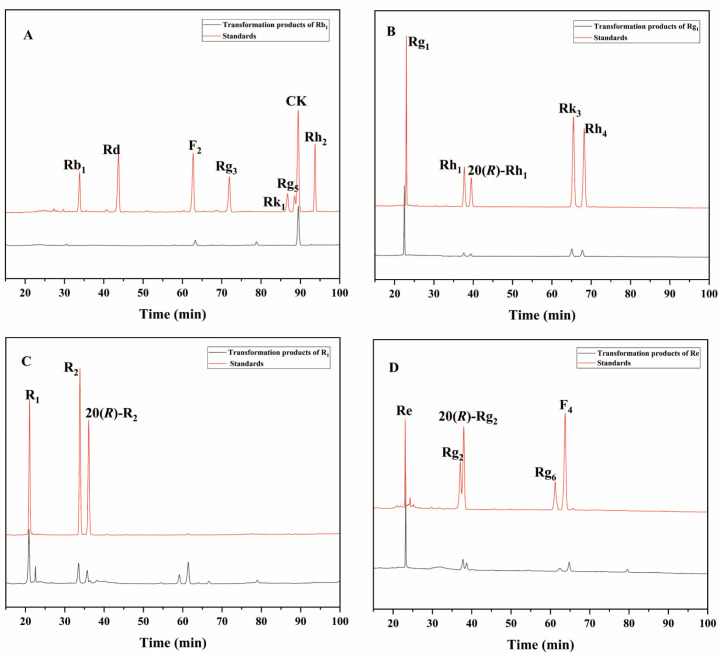
HPLC analysis of the transformation products of different types of saponins for 72 h by the crude enzyme from *P. fimorum*. (**A**) The transformation products of Rb_1_. (**B**) The transformation products of Rg_1_. (**C**) The transformation products of R_1_. (**D**) The transformation products of Re.

To further evaluate the enzyme’s ability to transform the total saponins of PNR, we qualitatively analyzed the products of the enzyme-mediated conversion of ginsenosides Rb_1_, Rg_1_, Re, and notoginseng R_1_ over 72 h by HPLC. The 72 h transformation products of the *P. fimorum* crude enzyme transforming ginsenoside Rb_1_ were found to have F_2_ and CK and an unknown product (Figure 7A). The transformation products of ginsenoside Rg_1_ included 20(*S*/*R*)-Rh_1_, Rk_3_, and Rh_4_ (Figure 7B). The transformation of notoginseng R_1_ yielded 20(*S*/*R*)-R_2_ (Figure 7C), while the transformation of ginsenoside Re produced 20(*S*/*R*)-Rg_2_, Rg_6_, and F_4_ (Figure 7D).

**Figure 8:**
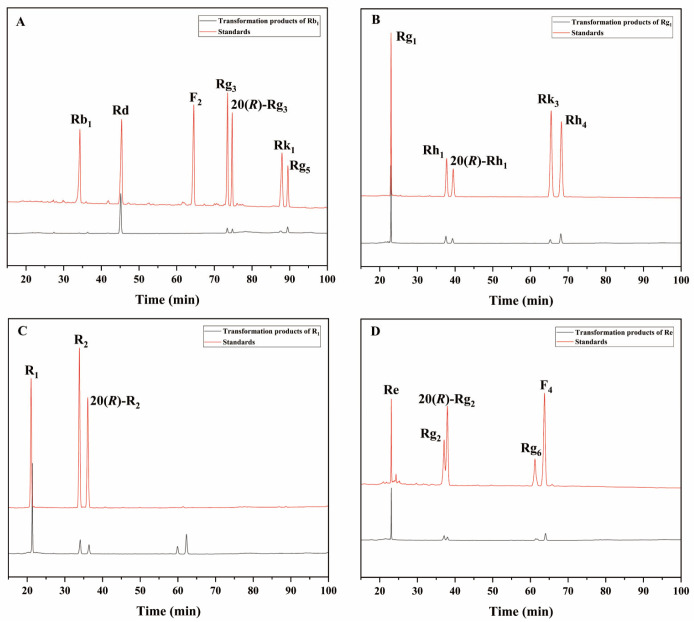
HPLC analysis of the transformation products of different types of saponins for 72 h by *β*-glucosidase. (**A**) The transformation products of Rb_1_. (**B**) The transformation products of Rg_1_. (**C**) The transformation products of R_1_. (**D**) The transformation products of Re.

The 72 h conversion products of commercial *β*-glucosidase transforming ginsenoside Rb_1_ had Rd, 20(*S*/*R*)-Rg_3_, Rk_1_, and Rg_5_ (Figure 8A); the 72 h conversion products of transforming ginsenoside Rg_1_ had 20(*S*/*R*)-Rh_1_, Rk_3_, and Rh_4_ (Figure 8B); and the 72 h conversion products of transforming notoginseng R_1_ had 20(*S*/*R*)-R_2_ (Figure 8C). The 72 h conversion products of transforming ginsenoside Re had 20(*S*/*R*)-Rg_2_, Rg_6_, and F_4_ (Figure 8D). The HPLC results of these two enzymes were generally consistent with the results of the TLC analyses.

### 3.4. Propose Possible Biotransformation Pathways of Major Ginsenosides Rg_1_, Re, Rb_1_, and Notoginsenoside R_1_ of PNR

**Figure 9:**
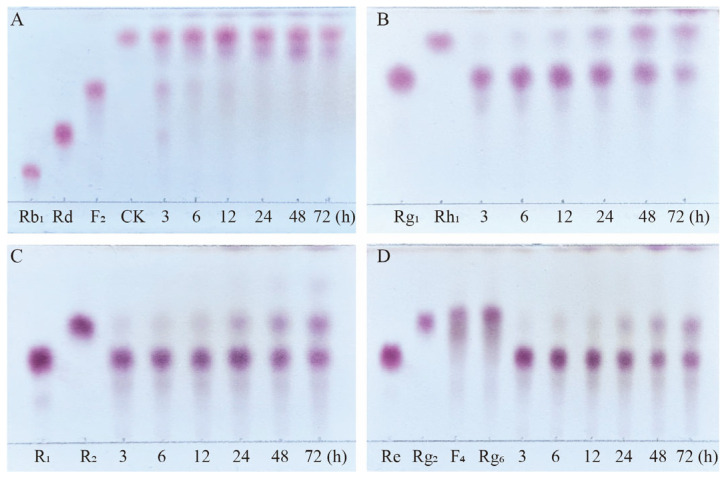
TLC analysis of the time-course variation in different types of saponins and their transformation products during the biotransformation process by the crude enzyme from *P. fimorum*. (**A**) Transformation products analysis of Rb_1_ dynamic monitoring. (**B**) Transformation products analysis of Rg_1_ dynamic monitoring. (**C**) Transformation products analysis of R_1_ dynamic monitoring. (**D**) Transformation products analysis of Re dynamic monitoring.

**Figure 10:**
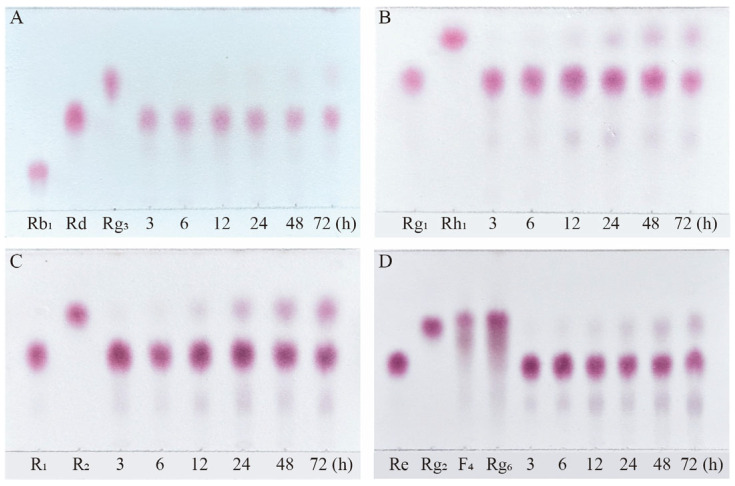
TLC analysis of the time-course variation in different types of saponins and their transformation products during the biotransformation process by *β*-glucosidase. (**A**) Transformation products analysis of Rb_1_ dynamic monitoring. (**B**) Transformation products analysis of Rg_1_ dynamic monitoring. (**C**) Transformation products analysis of R_1_ dynamic monitoring. (**D**) Transformation products analysis of Re dynamic monitoring.

**Figure 11:**
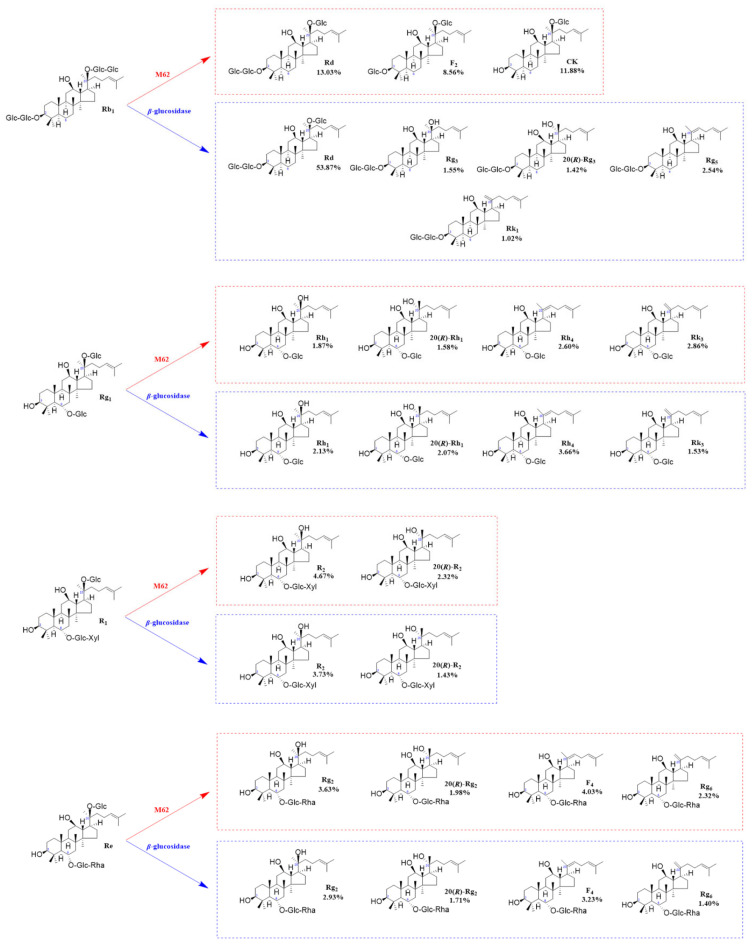
Structures and productivity of minor ginsenosides from the biotransformation of ginsenosides Rb_1_, Rg_1_, Re, and notoginseng R_1_ by crude enzyme from *P. fimorum* and *β*-glucosidase, respectively. Red dotted box: Products and yields of minor ginsenosides from biotransformation by the crude enzyme from *P. fimorum*. Blue dotted box: Products and yields of minor ginsenosides from biotransformation by *β*-glucosidase.

Based on qualitative and quantitative analyses by TLC and HPLC, as well as dynamic monitoring of the conversion reactions of several major saponins using linear regression equations (Table S1), we have proposed a pathway analysis for the conversion of the four major saponins in *P. notoginseng* by the crude enzyme from *P. fimorum* and commercial *β*-glucosidase. We also calculated the substrate conversion rates and product yields. Figure 9 presents the results of the dynamically monitored TLC analysis of the conversion of four major saponins by the *P. fimorum* crude enzyme, while Figure 10 shows the corresponding results for *β*-glucosidase. Table 1 summarizes the substrate conversion rates, and Figure 11 illustrates the proposed transformation pathway along with the yield analysis for each product.

### 3.5. Dynamic Change of Substrate Conversion of Major Ginsenosides and the Yield in the Transformation Products

**Figure 12:**
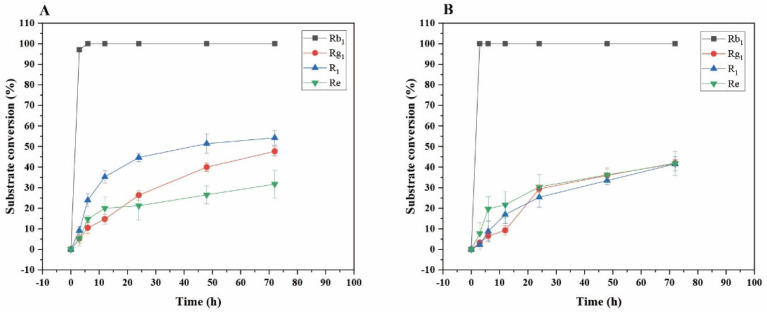
Dynamic changes in the substrate conversion of ginsenosides Rb_1_, Rg_1_, Re, and notoginsenoside R_1_ at different reaction times by the crude enzyme from *P. fimorum* (**A**) and *β*-glucosidase (**B**), respectively.

Transformation analysis of several major saponins and dynamic monitoring of product generation were performed. As shown in Figure 12A, for the transformation of ginsenoside Rb_1_ by the crude enzyme from *P. fimorum*, the substrate conversion rate reached its highest value of 100% at 6 h. For the transformation of ginsenoside Rg_1_, the highest transformation rate was 47.66% at 72 h; for notoginseng R_1_, the highest transformation rate was 54.23% at 72 h; and for ginsenoside Re, the highest transformation rate was 31.68% at 72 h.

As shown in Figure 12B, for the transformation of ginsenoside Rb_1_ by commercial *β*-glucosidase, the substrate transformation rate reached its highest value of 100% at 3 h. For the transformation of ginsenoside Rg_1_, the highest transformation rate was 41.94% at 72 h; for notoginseng R_1_, the highest transformation rate was 41.58% at 72 h; and for ginsenoside Re, the highest transformation rate was 41.72% at 72 h.

**Figure 13:**
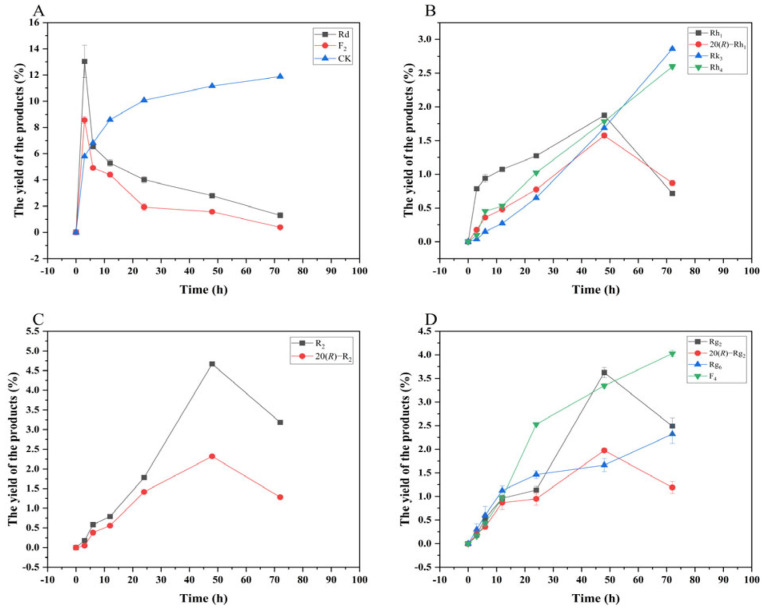
Dynamic changes in the yield of the transformation products of ginsenosides Rb_1_ (**A**), Rg_1_ (**B**), Re (**D**), and notoginsenoside R_1_ (**C**) at different reaction times by the crude enzyme from *P. fimorum*.

**Figure 14:**
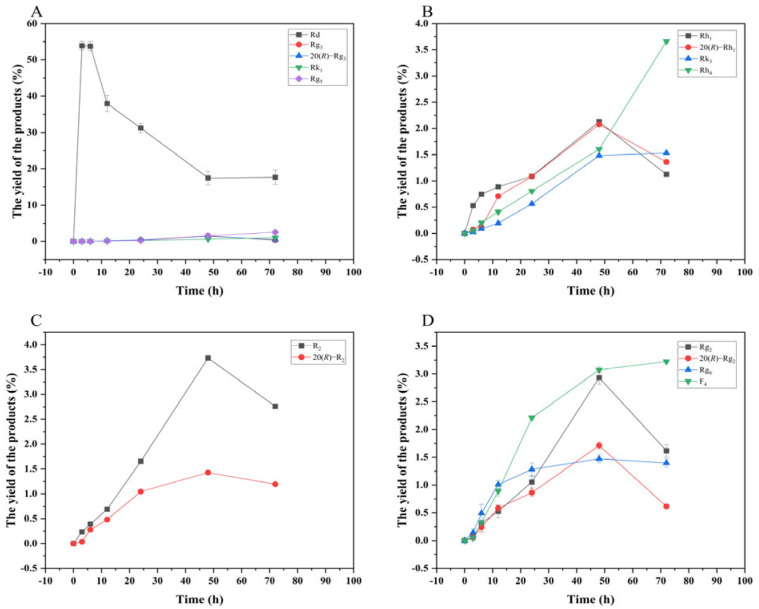
Dynamic changes in the yield of the transformation products of ginsenosides Rb_1_ (**A**), Rg_1_ (**B**), Re (**D**), and notoginsenoside R_1_ (**C**) at different reactions time by *β*-glucosidase.

Figure 13 shows the dynamic analysis results of the transformation product yields of four major saponins in *P. notoginseng* transformed by the crude enzyme from *P. fimorum,* while Figure 14 presents the corresponding results for the transformation by commercial *β*-glucosidase.

## 4. Discussion

In recent years, microbial transformation or biotransformation using enzymes have become a popular method for preparing minor ginsenosides, and many studies used these methods to transform the main ginsenosides for the preparation of minor ginsenosides [29,30,31,32].

In this study, *P. fimorum* was isolated from fresh *Gastrodia elata* and demonstrated the ability to transform the total saponins of PNR. The four major saponins (Rb_1_, Rg_1_, Re, and R_1_) from PNR were transformed using extracellular crude enzymes extracted from *P. fimorum*. The transformation products were thoroughly analyzed by TLC and HPLC. The transforming abilities of this fungus were found to include deglycosylation, epimerization, and dehydration, which, to the best of our knowledge, have not been previously reported for *P. fimorum*. The crude enzyme effectively hydrolyzed the glucose attached at the C-3 and C-20 positions of the major saponins, forming 20(*S*/*R*)-epimers at C-20 via isomerization, as well as double-bonded isomers at C-20 through dehydration.

According to most of the studies, it is known that *β*-glucosidase plays an important role in the conversion process against ginsenosides [26,27,29,30,31,32,33]. In this study, biotransformation experiments were conducted using commercial *β*-glucosidase to compare its transformation ability with that of the crude enzyme from *P. fimorum.* The results revealed significant differences in their transformation of ginsenoside Rb_1_. The crude enzyme from *P. fimorum* first hydrolyzed the glucose at the C-20 position of ginsenoside Rb_1_ to form ginsenoside Rd, and then sequentially hydrolyzed the glucose at the C-3 position of ginsenoside Rd, ultimately efficiently transforming it into ginsenoside CK. In contrast, *β*-glucosidase also formed ginsenoside Rd by hydrolyzing glucose at C-20 of ginsenoside Rb_1_, but then continued to hydrolyze it into 20(*S*/*R*)-Rg_3_, which further underwent dehydration to produce double-bonded isomers Rg_5_ and Rk_1_ at C-20. For the other three major saponins (Rg_1_, Re, and R_1_) the transformation abilities of commercial *β*-glucosidase and the crude enzyme from *P. fimorum* were nearly identical, as both enzymes efficiently transformed the major saponins into minor saponins.

Based on the analysis of transformation products by HPLC, several uncharacterized products were observed during the transformation of major saponins by the crude enzyme from *P. fimorum*. For example, for the transformation products F_2_ and CK of ginsenoside Rb_1_, there was an unknown product in the middle of them, while the transformation product 20(*S*/*R*)-R_2_ from notoginseng R_1_ was followed by several unknown products. Similarly, the transformation product F_4_ from ginsenoside Re was followed by an unknown product. These uncharacterized products may be new saponin derivatives, as their retention times closely resemble those of known minor saponins. If monomeric saponins can be efficiently transformed by the crude enzyme from *P. fimorum* in large quantities through fermentation and subsequently purified via column chromatography, there is potential to obtain novel saponin derivatives.

For the HPLC analysis of the conversion products of commercial *β*-glucosidase, we found that two unknown products were also present in the conversion products of notoginseng R_1_. By analyzing the transformation products of other major saponins, we can conclude the regularity that ginsenosides Rb_1_, Rg_1_, and Re can be transformed by *β*-glucosidase to form 20(*S*/*R*)-epimers, C-20(21) and C-20(22) double-bond isomers. Therefore, we hypothesize that notoginseng R_1_ can not only be transformed to 20(*S*/*R*)-R_2_, but it can be further transformed to the notoginsenoside T_5_ with a C-20(21) double bond, as well as 3*β*, 12*β*-Dihydroxydammarane-(*E*)-20(22), and 24-diene-6-O-*β*-D-xylopyranosyl-(1→2)-*β*-D-glucopyranoside with a C-20(22) double bond [4].

In addition, in this study, the transformation of various major saponins by the crude enzyme from *P. fimorum* and commercial *β*-glucosidase were monitored dynamically in terms of the conversion of substrates and the yield of the conversion products. The results of dynamic monitoring can thus be used for the qualitative production of a particular conversion product with a higher yield. Through the dynamic monitoring process, we know that the ginsenosides CK, F_2_, and F_4_ in the transformation products in this study have greater potential to be obtained through isolation and purification methods. The minor ginsenoside CK exhibits significant anticancer properties, particularly against hepatocellular carcinoma and breast cancer [34]. The minor ginsenoside F_2_ exhibits various biological activities, including antioxidant, anti-inflammatory, and anticancer properties [12]. The minor ginsenoside F_4_ can be used to treat type 2 diabetes mellitus [35].

In summary, this study investigated the transformation of major saponins from PNR using two enzymes, which is expected to improve the utilization of PNR and increase the methods of producing minor saponins.

## 5. Conclusions

In this experiment, biotransformation of the major saponins in PNR was carried out using the extracellular crude enzyme of the plant endophytic fungus *P. fimorum* and commercial *β*-glucosidase. The extracellular crude enzyme, extracted in our laboratory, could be further purified to enhance the transformation efficiency. Nevertheless, the biotransformation experiments demonstrated that the crude enzyme efficiently transformed ginsenoside Rb_1_ with a 100% substrate conversion rate, producing ginsenoside CK. While commercial *β*-glucosidase also achieved 100% conversion of ginsenoside Rb_1_, it generated multiple products, including Rd, 20(*S*/*R*)-Rg_3_, Rk_1_, and Rg_5_, which followed a different transformation pathway compared with the *P. fimorum* crude enzyme. For the total saponins in PNR, including notoginseng R_1_, ginsenoside Rg_1_, and Re, both the extracellular crude enzyme from *P. fimorum* and commercial *β*-glucosidase exhibited similar transformation abilities, transforming the major saponins into various minor saponins. This study also proposed a transformation pathway and analyzed the transformation rates and product yields through dynamic monitoring. Overall, the study introduces two methods for transforming major saponins in *P. notoginseng* into minor saponins, thereby improving the utilization of PNR and expanding the production pathways of minor saponins. These findings may be applicable to other parts of *P. notoginseng* and lay the groundwork for the development of genetically engineered strains and enzyme immobilization technologies for the large-scale production of minor saponins.

## Figures and Tables

**Table 1:** The substrate conversion of four main saponins from the roots of *P. notoginseng* during biotransformation.

The Types of Enzymes	Substrates	Substrate Conversion (%)
Crude enzyme from *P. fimorum*	Rb_1_	100
	Rg_1_	47.66
	R_1_	54.23
	Re	31.68
*β*-glucosidase	Rb_1_	100
	Rg_1_	41.94
	R_1_	41.58
	Re	41.72

## Data Availability

The original contributions presented in the study are included in the article/Supplementary Materials, further inquiries can be directed to the corresponding author.

## Supplementary Materials

The following supporting information can be downloaded at: https://www.mdpi.com/article/10.3390/microorganisms13030495/s1, Figure S1: The control experiment of the condition optimization process; Table S1: Linear regression equation of various saponins.
