# Supplementary material for: Comparison of the Transformation Ability of the Major Saponins in Panax notoginseng by Penicillum fimorum Enzyme and Commercial β-glucosidase
Source: Microorganisms. 2025 Feb 23;13(3):495. doi: 10.3390/microorganisms13030495 (PMC11944306; doi:10.3390/microorganisms13030495)
Supplement: Supplementary file 1 [file microorganisms-13-00495-s001.zip › microorganisms-3474373-supplementary.pdf]

Supporting Information

Comparison of the transformation ability of the major saponins in *Panax notoginseng* by *Penicillum fimorum* enzyme and commercial *β*-glucosidase

Feixing Li[1,†], Ruixue Zhang[1,†], Dongmei Lin[1], Jin Yang[1], Ye Yang[1,2], Xiuming Cui[1,2], Xiaoyan Yang[1,2,*]

[1] Faculty of Life Science and Technology, Kunming University of Science and Technology, Kunming 650500, China;

[2] Yunnan Key Laboratory of Sustainable Utilization of *Panax Notoginseng*, Kunming 650500, China

* Correspondence: yangxy@kust.edu.cn (X. Y.).

Tables of Contents

Figure S1. The control experiment of the condition optimization process.......................................3

Table S1. Linear regression equation of various saponins..3

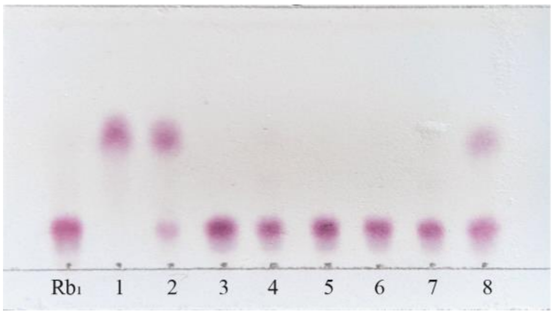

Figure S1. The control experiment of the condition optimization process. Rb_1: Substrate; 1-6: pH 3-8 control; 7 and 8: 60℃ and 70℃ control.

Table S1. Linear regression equation of various saponins.

Saponins	Regression eq	R^2	Linearity (mg/mL)
Rb_1	y = 5405.9x + 38.977	0.9997	0.023-0.75
Rd	y = 8338x + 40.517	0.9996	0.020n-0.65
F_2	y = 6202.1x + 22.247	0.9999	0.019-0.60
CK	y = 14771x + 56.428	0.9999	0.016-0.50
Rg_3	y = 4041.8x + 326.43	0.9991	0.028-0.90
20(*R*)-Rg_3	y = 4203.1x + 274.23	0.9992	0.025-0.80
Rk_1	y = 4837.9x + 43.247	0.9997	0.019-0.60
Rg_5	y = 10106x + 103.49	0.9997	0.022-0.70
R_1	y = 4678.2x + 106.3	0.9995	0.019-0.60
R_2	y = 11672x + 18.398	0.9991	0.017-0.55
20(*R*)-R_2	y = 3242x + 43.4	0.9996	0.019-0.60
Rg_1	y = 6967.6x + 65.27	0.9991	0.016-0.50
Rh_1	y = 7323.7x + 105.69	0.9993	0.017-0.55
20(*R*)-Rh_1	y = 6099.3x + 75.74	0.9993	0.016-0.50
Rk_3	y = 20379x + 301.41	0.9992	0.017-0.55
Rh_4	y = 16346x + 251.37	0.9994	0.019-0.60
Re	y = 1659.8x + 75.114	0.9991	0.019-0.60
Rg_2	y = 3250.2x + 87.499	0.9993	0.017-0.55
20(*R*)-Rg_2	y = 5142.9x + 170.84	0.9994	0.019-0.60
Rg_6	y = 2308.6x + 66.824	0.9992	0.017-0.55
F_4	y = 6824.9x + 218.2	0.9993	0.019-0.60
